# Elastic Relaxation of Coherent InGaN/GaN Interfaces at the Microwire LED Sidewall

**DOI:** 10.1002/advs.202408736

**Published:** 2025-02-26

**Authors:** Jongil Kim, Jinwook Yeo, Bumsu Park, Jeehun Jeong, Seunghwa Ryu, Sang Ho Oh

**Affiliations:** ^1^ Department of Energy Engineering Institute of Energy Materials and Devices Korea Institute of Energy Technology (KENTECH) Naju 58330 Republic of Korea; ^2^ Department of Mechanical Engineering Korea Advanced Institute of Science and Technology (KAIST) Daejeon 34141 Republic of Korea; ^3^ CEMES‐CNRS 29 rue. J. Marvig Toulouse 31055 France

**Keywords:** GaN, interface, light emitting diodes, microwire, strain, transmission electron microscopy

## Abstract

Elastic relaxation of lattice misfit strain via traction‐free surface results in complex 3D strain distribution and morphological modification at the boundary of epitaxial heterostructure. While this phenomenon is extensively studied, the influence of the interface coherency constraining the strain relaxation has received little attention. Here it is shown that the interfacial shear stresses arise toward the traction free sidewall of microscale light emitting diode (LED) wires while the two complementary strained InGaN and GaN layers are relaxed to revert their bulk lattice parameters near the sidewall. The shear stresses with opposite signs achieve mechanical equilibrium by counterbalancing the change in the sign of the in‐plane strain in each layer of the near‐surface region. A unique nonmonotonic modulation of both normal and shear strain is detected unambiguously in the strain maps and corroborated by finite element modeling. An analytical model is developed based on the Airy stress function, which incorporates the superposition of alternating in‐plane pre‐stress and the image stress to satisfy the boundary condition. The resultant complex strain fields in microscale LEDs, where surface emission is dominant, alter strain‐induced piezoelectric polarization near the surface, significantly affecting electro‐optical efficiency and resulting in spectral broadening and/or wavelength shifts in emitted light.

## Graphical abstract

Strain mapping and modeling reveal that the interfacial shear stresses arise toward the traction free sidewall of microscale light emitting diode (LED) wires while the two complementary strained InGaN and GaN layers in multiple quantum wells are relaxed to revert their bulk lattice parameters near the sidewall.

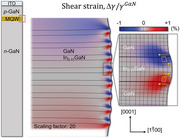

## 1 Introduction

In_x_Ga_1‐x_N (InGaN)/GaN multiple quantum wells (MQWs) serve as the primary active layer in blue and green light‐emitting diodes (LEDs). In these structures, the InGaN quantum wells (QWs) are typically strained to maintain lattice coherence with the GaN quantum barriers (QBs). This elastic strain induces a piezoelectric polarization along the growth direction of the InGaN QWs, resulting in a net polarization that is the sum of the strain‐induced piezoelectric polarization and the spontaneous polarization.^[^ ^1^, ^2^ ^]^ A polarization mismatch between the InGaN QWs and GaN QBs leads to bound charges of alternating signs at the MQW interfaces, creating an internal electric field within the InGaN QWs.^[^ ^3^ ^]^ In conventional blue LEDs, this polarization field generated in a few nm‐thick InGaN QW layer typically reaches several MV cm^−1^,^[^ ^1^, ^4^, ^5^ ^]^ which has been regarded as a major factor contributing to the degradation of internal quantum efficiency (IQE). Moreover, this polarization field increases with higher In content, posing a challenge for the development of longer wavelength devices based on a polar InGaN/GaN MQWs structure.

Extensive efforts have been made to mitigate the strain‐induced piezoelectric polarization through strain engineering,^[^ ^6^, ^7^ ^]^ but complete elimination of this effect in polar InGaN QWs remains difficult. Instead, approaches such as patterning the sapphire substrate, introducing controlled superlattice (SL) layers, or adding InGaN strain‐relief layers have been explored to enhance the optical efficiency of standard InGaN/GaN MQWs based LEDs.^[^ ^8^, ^9^, ^10^, ^11^, ^12^ ^]^ The key to these strategies is increasing the density of V‐pit defects, which effectively reduce the negative impact of threading dislocations on optical efficiency.^[^ ^11^, ^12^ ^]^ Another approach to overcome strain‐induced issues is to alter the growth orientation of InGaN/GaN MQWs from the polar *c*‐plane to non‐polar orientations, such as $\left(10\bar{1}0\right)$ *m*‐plane or $\left(11\bar{2}0\right)$ *a*‐plane.^[^ ^13^, ^14^ ^]^ In these configurations, the polarization lies parallel to the InGaN/GaN interface plane. However, the peak IQE of conventional non‐polar and semi‐polar InGaN LEDs consistently falls short of that achieved by standard polar (0001)‐oriented LEDs.^[^ ^15^ ^]^

Recently, the pursuit of high pixel density displays has catalyzed the advancement of microscale and nanoscale LEDs, commonly referred to as µLEDs and nLEDs, respectively.^[^ ^16^, ^17^ ^]^ Typically, one‐dimensional rod‐like form of µLED is fabricated through a top‐down etching process applied to standard planar InGaN/GaN MQW heterostructure. As the surface‐to‐volume ratio increases with miniaturization, the properties of µLEDs become increasingly influenced by surface defects and strain states.^[^ ^18^, ^19^ ^]^ The chemical and ion etching processes integral to µLED array fabrication often introduce non‐radiative defects along the sidewalls, necessitating process optimization to minimize defect formation and subsequent surface passivation to remedy these defects.^[^ ^20^ ^]^

Beyond these fabrication challenges, µLEDs, especially within the InGaN/GaN MQW region, confront an intrinsic challenge: elastic relaxation of the misfit strain via the traction‐free sidewalls.^[^ ^19^, ^21^, ^22^ ^]^ This strain relaxation has been recognized as beneficial in µLEDs, enabling the accommodation of greater misfit strain from higher indium concentrations while reducing piezoelectric polarization fields.^[^ ^23^ ^]^ However, it also leads to a complex three‐dimensional (3‐D) strain distribution within the small volume of heterostructure and causes morphological modification of the MQW at the sidewall.^[^ ^23^, ^24^, ^25^ ^]^ The strain‐induced piezoelectric polarization and the resultant electric fields lead to spectral broadening or peak shifts in the light emitted from near‐surface InGaN quantum wells (QWs).^[^ ^9^, ^11^, ^21^ ^]^ Nonetheless, the mechanical boundary conditions imposed on the coherent InGaN/GaN interfaces at the µLED sidewall – strain relaxation while maintaining lattice coherency – have yet to be thoroughly investigated in mechanical modeling or microscopic characterization.^[^ ^19^, ^24^, ^26^, ^27^ ^]^

The present study employs high‐resolution scanning transmission electron microscopy (STEM) strain mapping to investigate the elastic strain relaxation near a µLED sidewall. The experimental strain maps show that the lattice coherency persists throughout the MQW heterostructure, from core to sidewall. In the central region of the µLED, the InGaN and GaN lattices are matched complementarily under in‐plane prestress conditions, with the former under compression and the latter tension, a phenomenon analogous to the strain partitioning in a free‐standing epitaxial bilayer films.^[^ ^28^ ^]^ However, toward the traction‐free sidewall, both layers are relaxed to revert their bulk lattice parameters. This transition induces a gradual increase in shear stress, which achieves mechanical equilibrium by counterbalancing the change in the sign of the in‐plane strain. A unique nonmonotonic modulation of both normal and shear stresses in the near‐surface region of InGaN/GaN MQW has been detected unambiguously in the experimental strain maps and further corroborated by finite element modeling (FEM). The nonmonotonic stress modulation observed near the sidewall was elucidated by an analytical model that employs the Airy stress function, taking into account the superposition of a bulk multilayer structure subjected to alternating in‐plane pre‐stress and image stress to adhere to the traction‐free sidewall boundary condition. The strain distribution identified in this study is likely prevalent in other nanostructured epitaxial heterostructures with substantial free surface portions, underscoring its importance in accurately understanding surface strain distribution.

## 2 Results and Discussion

### 2.1 InGaN/GaN MQW Heterostructure in µLED

**Figure 1:**
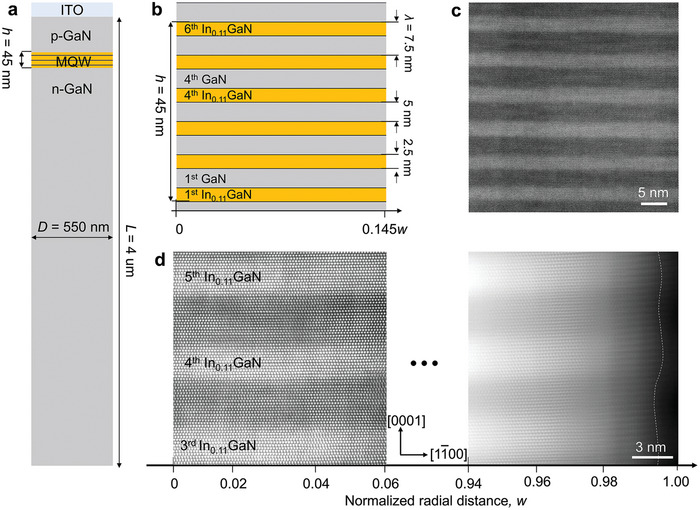
In_0.11_GaN/GaN µLED structure, its elastically strained (center), and relaxed (sidewall) atomic lattices of MQW. a) Schematic illustration of model µLED system. The wire diameter (*D* = 550 nm) and length (*L* = 4 µm) and the thickness of six pairs of InGaN/GaN MQW (*h* = 45 nm) are indicated. b) Schematic illustration of the InGaN/GaN MQW heterostructure. Each InGaN QW and GaN QB is 2.5 and 5 nm in thickness, respectively, together constituting a 7.5 nm‐thick repeating period (λ). The height‐to‐diameter ratio, *h*/*D*, is 0.08. c) HAADF STEM image of the InGaN/GaN MQW. d) High‐magnification HAADF STEM images of the central region (*w* = 0) and near the sidewall (*w* = 1). The lattice coherency is maintained across the entire InGaN/GaN interface region from the center to the sidewall. The severe bending of the out‐of‐plane lattice is observed near the sidewall from ≈0.98 *w* where the elastic strain is relaxed. Note that the STEM image in d) was obtained from the suspended wire sample without thinning by FIB.

A regular array of µLED pattern with the wire diameter (*D*) of ≈550 nm was fabricated through photolithography followed by reactive ion etching. Subsequently, potassium hydroxide (KOH) wet etching was performed to remove the sidewall damages caused by the plasma during the reactive ion etching. These top‐down‐processed µLEDs consist of an indium tin oxide (ITO)/*p*‐GaN/MQW/*n*‐GaN structure (**Figure** **1**a). The six pairs of In_0.11_GaN/GaN MQWs were grown epitaxially on *n*‐GaN/sapphire substrate without noticeable plastic strain relaxation by defects. The thickness of each InGaN QW and GaN QB is 2.5 and 5 nm, respectively, together constituting a 7.5 nm‐thick repeating period (λ) of InGaN/GaN MQW (Figure 1b). The thickness‐to‐diameter ratio of the heterostructure, *h* */* *D*, where *h* is the thickness of the total six pairs of InGaN/GaN MQW, is 45/550 ≈ 0.08. All InGaN/GaN interfaces in the MQW heterostructure are perfectly coherent devoid of misfit dislocations (Figure 1c,d).

The internal strain fields in the MQW heterostructure in a µLED depend critically on the *h* */* *D* ratio.^[^ ^29^ ^]^ When *h* << *D*, the system is close to the planar heterostructure deposited on a semi‐infinite substrate and the elastic strain is essentially the same as in the laterally infinite system except at the very edge of the µLED. As *h* increases, the strained epi‐layer (InGaN) starts relaxing the strain from the sidewalls and the GaN QB and InGaN QW share the strain even though the GaN substrate is semi‐infinite. For *h* */* *D* = 0.08 as in the present case, we confirmed that the misfit strain of InGaN QW is shared slightly with GaN QB even at the center of µLED (Figures S1a and S2b, Supporting Information). The lattice matched in‐plane lattice parameter ($a_{0}$) calculated by applying the zero‐stress criterion is 0.319 nm,^[^ ^30^ ^]^ which lies in‐between that of GaN ($a_{\mathrm{GaN}}$ = 0.318 nm) and InGaN ($a_{\mathrm{InGaN}}$ = 0.322 nm) but closer to the former. As a consequence of the strain partitioning, the out‐of‐plane strain due to the Poisson's effect deviates from one that is expected for a planar heterostructure;^[^ ^28^, ^31^ ^]^ while the GaN QB is compressed due to the in‐plane tensile strain, the InGaN QW is expanded less compared to the planar heterostructure due to the smaller in‐plane compressive strain (Figures S1b and S2b, Supporting Information). This in‐plane and out‐of‐plane strain state persists almost up to 90% of the normalized radial distance.^[^ ^19^ ^]^ The strain maps and profiles are presented as a function of the radial distance normalized by the wire radius, *w*, in all figures.

### 2.2 Elastic Strain Relaxation via Traction‐Free Surface

The strained InGaN and GaN lattices start relaxing toward their bulk state roughly around from 0.9 *w*.^[^ ^19^ ^]^ It is important to note that the interface coherency is preserved almost to the sidewall (i.e., free surface) while the strain in each layer is relaxed. Due to the lattice coherency, the elastic relaxation is constrained by the in‐plane lattices which are strongly clamped at the interfaces and resist the relaxation.^[^ ^19^, ^29^ ^]^ The high‐resolution STEM image of the MQW sidewall reveals distinct lattice fringes appearing from ≈0.98 *w*, where the out‐of‐plane lattice planes bend severely (Figure 1d). Furthermore, this interface‐constrained elastic relaxation results in the morphological change of sidewall to a wavy shape with crest at the InGaN and trough at the GaN (refer to the dash line depicting the sidewall morphology in Figures 1d).

**Figure 2:**
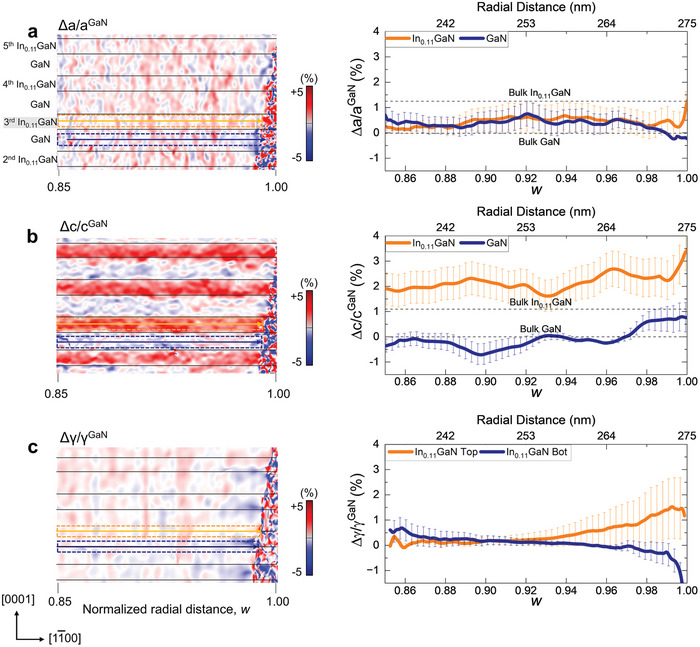
STEM GPA strain maps and profiles of the InGaN/GaN MQW region near the µLED sidewall. a) In‐plane, c) out‐of‐plane, and c) shear strain map of 4 pairs of InGaN QW and GaN QB from 0.85 to 1.0 *w*. The profiles of strain were obtained from the 3^rd^ InGaN QW (orange dashed box) and the 3^rd^ GaN QB (blue dashed box). The strain relaxation starts gently from 0.85 *w* toward the bulk values. An abrupt change in the elastic strain relaxation is observed at ≈0.98–0.99 *w*. Note that the strain was calculated by using the bulk lattice parameter of GaN (*a*_GaN_) as reference. The noisy fluctuations originate from a finite background noise of the GPA strain map.

**Figure 3:**
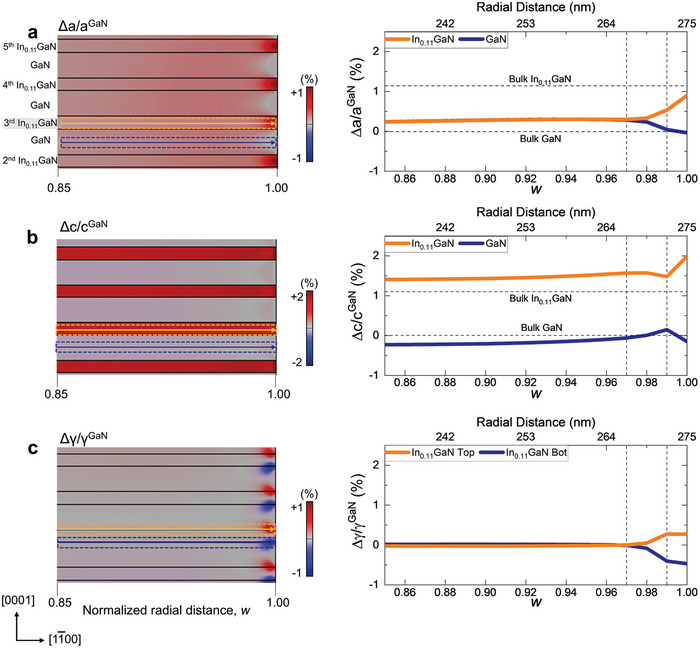
FEM strain maps and profiles of the InGaN/GaN MQW region near the µLED sidewall. a) In‐plane, b) out‐of‐plane c) shear strain map of 4 pairs of InGaN QW and GaN QB from 0.85 to 1.0 *w*. The strain maps were obtained from the same area as in Figure 2. The profiles of strain were obtained from the 3^rd^ InGaN QW (orange dashed box) and the 3^rd^ GaN QB (blue dashed box). In consistent with the experimental strain results, an abrupt change in the elastic relaxation is observed at ≈0.98–0.99 *w*. Opposite sign of interfacial shear strain is induced and exhibits the same trend as in the experimental result, which is the key for maintaining the lattice coherency during elastic relaxation.

To investigate the interface‐constrained strain relaxation behavior of each layer in the MQWs, the strain profiles were obtained separately from the InGaN QW and the GaN QB near the sidewall (0.85 < *w <* 1.0) (**Figure** **2**) and compared with those obtained by FEM (**Figure** **3**). The FEM simulation perfectly reproduces the key features of the experimental strain profiles, validating the FEM‐assisted interpretation of the experimental strain data. While the two layers maintain the lattice coherency, i.e., the same in‐plane lattice parameter of $a_{0}$, (Figures 2a and 3a), the out‐of‐plane strain starts relaxing gently already from 0.85 *w* toward the bulk value (Figures 2b and 3b). This trend changes abruptly very close to the sidewall (≈0.98–0.99 *w*), where the strain of the two layers diverges to different values. Moreover, the shear strain with opposite sign arises in each layer in this regime (Figures 2c and 3c). We note that the noisy fluctuations in the experimental strain profiles originate from a finite background noise level of the geometrical phase analysis (GPA) strain map, which is ≈±0.1%.

The radial (in‐plane) strain profiles obtained from the InGaN QWs and adjacent GaN QBs reveal that the in‐plane lattice‐matched condition persists up to approximately 0.98 *w*. Note that the two lattices remain coherent at the interface while the strain is relaxed in each layer. The in‐plane matched lattice parameter ($a_{0}$) measured from the experiment and FEM is 0.3185 and 0.3181 nm, respectively. These values are smaller but comparable to the calculated value of 0.319 nm obtained by applying the zero‐stress criterion.^[^ ^30^ ^]^ Notably, from ≈0.98 *w*, the in‐plane lattice parameters begin diverging toward their respective bulk values, a phenomenon attributable to the traction‐free conditions present at the sidewall.

**Figure 4:**
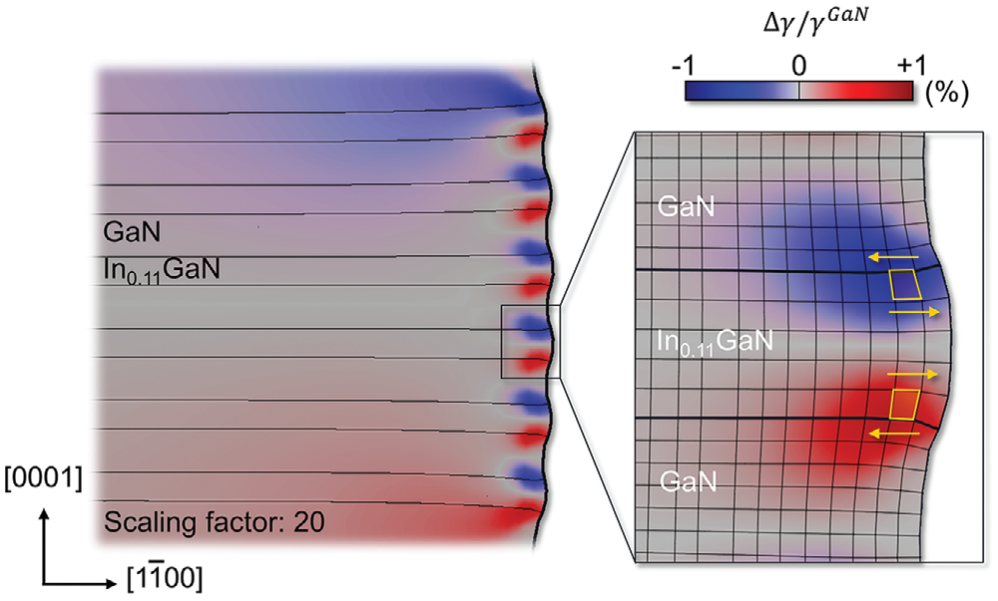
FEM shear strain map and corresponding deformation mesh near the surface of µLED sidewall. As a result of the in‐plane stress relaxation through the sidewall, shear strain emerges at both the upper and lower InGaN/GaN interfaces of each In_0.11_GaN layer but with opposite signs, facilitating elastic relaxation and inducing volume expansion of the In_0.11_GaN layer.

In the central region of the µLED, the axial (out‐of‐plane) strain of each layer follows what is expected from the Poisson effect of the non‐zero in‐plane strain (Figures S1b and S2b, Supporting Information).^[^ ^19^ ^]^ However, as one approaches the sidewall the strain deviates from this trend. From 0.9 to 0.98 *w*, the out‐of‐plane strain in both the InGaN QWs and GaN QBs progressively increases. At 0.99 *w*, the out‐of‐plane strain in each layer takes different path; the out‐of‐plane lattice parameter of the GaN QB contracts to its bulk value, while that of the InGaN QW expands beyond its bulk value. This behavior indicates a volumetric expansion of the InGaN layer near the sidewall, as illustrated in **Figure** **4**.

Another notable feature in the strain distribution is the emergence of shear strain near the sidewall, a feature does not exist in the central region (Figure 4). The shear strain, occurring at both the upper and lower interfaces of each layer but with opposite signs, acts to counterbalance the elastic relaxation within each layer while preserving the lattice coherency, as demonstrated by the analysis of STEM image in Figure S3 (Supporting Information). According to the FEM simulations, shear strain begins to arise at the point where the in‐plane strain relaxation initiates (Figure 3c, ≈0.98 *w*). Experimentally, however, shear strain arises at a location further away from the sidewall (Figure 2c, ≈0.94 *w*). This broader distribution of shear strain is likely influenced by structural imperfections or surface roughness of the sidewall introduced during the etching processes.^[^ ^22^, ^32^ ^]^

As indicated by two vertical dash lines in Figure 3, the FEM simulation identified the characteristic strain fields induced by interface‐constrained elastic relaxation over two distinctive length scales from the sidewall. First, a unique monotonic fluctuation in in‐plane, out‐of‐plane, and shear strain initiates when the radial distance approaches the sidewall approximately within the structural periodicity (λ = 7.5 nm ≈0.03 *w*) at 0.97 *w*. This strain field conforms to the conventional expectation that the characteristic length scale of strain relaxation from the surface is related to the defect size or loading periodicity.^[^ ^33^ ^]^ Second, in the region very close to the sidewall, i.e., 0.99 *w*, the strain field deviates from the expected relaxation trend and undulates rapidly.

### 2.3 Mechanical Modelling

Having validated the characteristic strain fields near the surface through both experimental observation and simulation, we next explored the stress fields which cause the observed strain fields adjacent to the sidewall. In the absence of the sidewall, the stress fields within a planar MQW heterostructure, comprising alternating infinite layers of InGaN and GaN, are primarily dictated by the lattice misfit strain between these materials and their respective bulk moduli. Assuming that the radial pre‐strain of InGaN and GaN, parallel to the coherent interface, is represented by $\varepsilon_{11,\mathrm{InGaN}}^{0}$ and $\varepsilon_{11,\mathrm{GaN}}^{0}$ for infinite domains, respectively, and their elastic moduli and the thicknesses are denoted as $E_{\mathrm{InGaN}}$, $E_{\mathrm{GaN}}$, $t_{\mathrm{InGaN}}$ and $t_{\mathrm{GaN}}$, respectively, mechanical equilibrium necessitates that the stress state adheres to the following equation:

(1) $$E_{\mathrm{InGaN}}\varepsilon_{11,\mathrm{InGaN}}^{0}t_{\mathrm{InGaN}}+E_{\mathrm{GaN}}\varepsilon_{11,\mathrm{GaN}}^{0}t_{\mathrm{GaN}}=0$$

Here, the radial pre‐strain values of two layers are denoted as $\varepsilon_{11,\mathrm{InGaN}}^{0}=\left(a_{0}-a_{\mathrm{InGaN}}\right)/a_{\mathrm{InGaN}}$ and $\varepsilon_{11,\mathrm{GaN}}^{0}=\left(a_{0}-a_{\mathrm{GaN}}\right)/a_{\mathrm{GaN}}$, where $a_{\mathrm{InGaN}}$ and $a_{\mathrm{GaN}}$ are the lattice constant of InGaN and GaN, respectively. The InGaN layer undergoes compressive deformation ($\varepsilon_{11,\mathrm{InGaN}}^{0}<0$) while the GaN layer experiences tensile deformation ($\varepsilon_{11,\mathrm{GaN}}^{0}>0$). To estimate the distribution of axial strain ($\varepsilon_{22}^{0}$) within each layer, we used the radial pre‐strain and Poisson's ratio (v) based on the linear elasticity theory. This relationship is approximated as $\varepsilon_{22}=-2v\varepsilon_{11}$.^[^ ^34^ ^]^ Subsequently, the pre‐radial stress ($\sigma_{11}^{0}$) and pre‐axial stress ($\sigma_{22}^{0}$) can be calculated under the assumption of plane stress conditions, using the following formulas: $\sigma_{11}^{0}=E/\left(1-v^{2}\right)\left(\varepsilon_{11}^{0}+v\varepsilon_{22}^{0}\right)$ and $\sigma_{22}^{0}=E/\left(1-v^{2}\right)\left(v\varepsilon_{11}^{0}+\varepsilon_{22}^{0}\right)$, respectively. There exists no shear stress in the bulk MQW heterostructure.

If the uniform stress distribution is maintained within the µLED domain Ω, then the traction on the sidewall surface ∂Ω must exist, represented as $F=\sigma^{0}\cdot n$. However, due to the zero‐traction (or free surface) on the sidewall, a compensating image stress $\sigma^{S}$ is required to negate the surface traction. Hence, the traction associated with the image stress must be given as $T^{S}=-F=\sigma^{0}\cdot n$. Since the sign of $\sigma^{0}$ acting in the InGaN and GaN layers is opposite, the effect of the image stress is equivalent to superimposing periodic negative traction on the sidewall, which ultimately leads to the alternating shear stress and variation of radial and axial stress near the surface.

**Figure 5:**
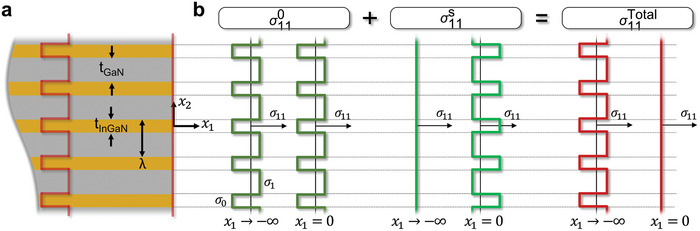
Mechanical modeling of the image stress. a) Geometry of continuum model and b) boundary condition for the radial stress. For simplicity, we considered a half‐space and infinite array. λ is the period of repeating unit (i.e., $\lambda =t_{\mathrm{InGaN}}+t_{\mathrm{GaN}}$), $\sigma_{11}^{0}$ is the pre‐radial stress, $\sigma_{11}^{S}$ is the image radial stress, and $\sigma_{11}^{Total}$ is the total radial stress including the pre‐stress and the image stress.

In order to elucidate the stress fields induced by surface effects in relation to the image stress, a streamlined continuum model was established, from which an analytical solution was derived. To simplify the analysis, we assume both materials to be elastically isotropic and identical except their lattice constants, yet the qualitative strain distribution characteristics near the sidewall are still properly captured. Adopting a coordinate system with its origin at the midway point of an InGaN layer on the sidewall, where $x_{1}$ and $x_{2}$ represents the radial direction and the axial direction, respectively (**Figure** **5**a), we are able to illustrate the radial component of normal stress both at the surface and at deeper positions, as depicted in Figure 5b. For simplicity, we considered a half‐space and infinite array, as our primary objective is to reveal the effect of traction‐free sidewall on the elastic relaxation of complementary strain in the epitaxial layers. As to the distribution of stress for bulk heterostructure, the radial pre‐stress ($\sigma^{0}$) can be expressed as follows,

(2) $$\sigma^{0}\left(x_{2}\right)=\sigma^{0}\left(x_{2}+\lambda \right)$$

(3) $$\sigma_{\mathrm{ij}}^{0}=\left\{\begin{array}{c} \sigma_{\mathrm{ij},\mathrm{InGaN}}^{0}\cdots \left(-0.5t_{\mathrm{InGaN}}<x_{2}<0.5t_{\mathrm{InGaN}}\right)\\ \sigma_{\mathrm{ij},\mathrm{GaN}}^{0}\cdots \left(0.5t_{\mathrm{InGaN}}<x_{2}<0.5t_{\mathrm{InGaN}}+t_{\mathrm{GaN}}\right) \end{array}\right\}$$

where λ is the period of repeating unit (i.e., $\lambda =t_{\mathrm{InGaN}}+t_{\mathrm{GaN}}$). On the other hand, the radial component of the image stress $\sigma_{11}^{S}\left(x_{1},x_{2}\right)$ should decay to zero in the limit of $x_{1}\to -\infty$, while at the surface ($x_{1}=0$), it cancels out the bulk stress by $\sigma_{11}^{S}\left(0,x_{2}\right)=-\sigma_{11}^{0}\left(0,x_{2}\right)$. The corresponding Airy stress function $\phi^{S}(x_{1},x_{2})$ for the surface‐induced stress which satisfies the compatibility equation $\nabla^{4}\phi (x_{1},x_{2})=0$ as well as the prescribed boundary condition, can be obtained as follows,

(4) $$\phi^{S}\left(x_{1},x_{2}\right)=\sum_{n=1}^{\infty }A_{n}\cos\left(\frac{2n\pi }{\lambda }x_{2}\right)\left(1-\frac{2n\pi }{\lambda }x_{1}\right)e^{\frac{2n\pi }{\lambda }x_{1}}$$

where $A_{n}=-\frac{\lambda^{2}}{2n^{3}\pi^{3}}\sin\left(\frac{n\pi }{\lambda }t_{InGaN}\right)\left(\sigma_{11,InGaN}^{0}-\sigma_{11,GaN}^{0}\right)$.

From the Airy function, the stress components are determined as $\sigma_{11}=\partial^{2}\phi /\partial x_{2}^{2}$, $\sigma_{22}=\partial^{2}\phi /\partial x_{1}^{2}$, $\sigma_{12}=-\partial \phi \partial x_{1}\partial x_{2}$, in a way to satisfy mechanical equilibrium. Accordingly, the surface‐induced stress components can be obtained as follows,

(5) $$\sigma_{11}^{S}\left(x_{1},x_{2}\right)=\sum_{n=1}^{\infty }B_{n}\cos\left(\frac{2n\pi }{\lambda }x_{2}\right)\left(1-\frac{2n\pi }{\lambda }x_{1}\right)e^{\frac{2n\pi }{\lambda }x_{1}}$$

(6) $$\sigma_{22}^{S}\left(x_{1},x_{2}\right)=\sum_{n=1}^{\infty }B_{n}\cos\left(\frac{2n\pi }{\lambda }x_{2}\right)\left(1+\frac{2n\pi }{\lambda }x_{1}\right)e^{\frac{2n\pi }{\lambda }x_{1}}$$

(7) $$\sigma_{12}^{S}\left(x_{1},x_{2}\right)=\sum_{n=1}^{\infty }B_{n}\frac{2n\pi }{\lambda }\sin\left(\frac{2n\pi }{\lambda }x_{2}\right)x_{1}e^{\frac{2n\pi }{\lambda }x_{1}}$$

where $B_{n}=\frac{2}{n\pi }\sin\left(\frac{n\pi }{\lambda }t_{\mathrm{InGaN}}\right)\left(\sigma_{11,\mathrm{InGaN}}^{0}-\sigma_{11,\mathrm{GaN}}^{0}\right)$.

The resulting stress field $\sigma^{S}$ can be considered as a superposition of wavy stress distributions with their periodicity of λn, where n is integer. Their amplitude decays exponentially from the surface with the characteristic length scale of λn.

**Figure 6:**
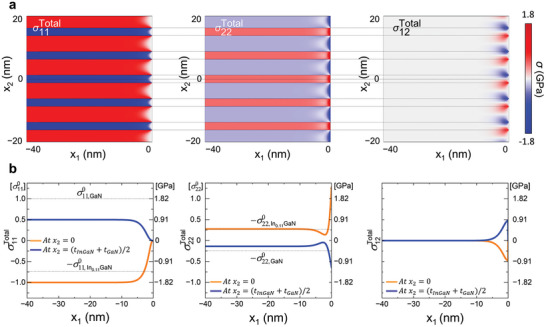
Stress fields calculated from mechanical model. a) Total stress fields and b) profiles which are the sum of the pre‐stress and image stress, i.e., $\sigma^{\mathrm{Total}}=\sigma^{0}+\sigma^{S}$. The stress profile extracted from the center of InGaN ($x_{2}=0$) and GaN ($x_{2}=(t_{\mathrm{InGaN}}+t_{\mathrm{GaN}})$) layer is displayed in orange and blue line, respectively. To satisfy in‐plane stress free condition due to the nature of free surface of microwire, the radial stress component $\sigma_{11}^{Total}$ exhibits surface‐induced effects within the characteristic length λ = 7.5 nm from the surface. As a result, the axial stress of the opposite sign appears to relax, and a shear stress of the opposite sign occurs to maintain lattice registry in the heterostructure.

As the largest component $A_{n}$ coincides with the primary periodicity λ (i.e., n = 1), and other high‐order components being more rapidly decaying from the surface, the normal stress components $\sigma_{11}^{Total}$ and $\sigma_{22}^{Total}$ exhibit surface‐induced effects within the characteristic length λ from the surface as shown in **Figure** **6**a,b, where $\sigma^{\mathrm{Total}}=\sigma^{0}+\sigma^{S}$. In fact, closer examination of both experimental and FEM strain maps reveal that the surface‐induced relaxation becomes effective already from a distance comparable to λ (7.5 nm = 0.03 *w*) from the surface. The characteristic length scale λ dictates an exponential decay of stress approximately to 36% (∼1/e). However, nonmonotonic change in the axial stress $\sigma_{22}^{Total}$ is found across λ. This quicker andnonmonotonic alteration near the surface is attributed to the effect of multiplicative prefactor (1+(2π/2πλλ)x1). Moreover, the axial normal stresses decrease slightly and then amplify near the surface, primarily resulting from the image stress $\sigma^{S}$ counterbalancing the traction from $\sigma^{0}$. For additional details on the prefactor, refer to the supplementary information (Figure S4, Supporting Information).

The amplified axial normal stress $\sigma_{22}^{Total}$ results in the volume expansion, especially in the InGaN QW layers. Intriguingly, the shear stress component, which is absent in $\sigma^{0}$, emerges near the surface as shown in $\sigma_{12}^{Total}$ of Figure 6a,b. To facilitate surface‐induced image stress $\sigma^{S}$, featuring periodic traction with a periodicity of λ at the surface and internal stress decaying exponentially within the distance of λ, the interfacial shear stress must develop near the surface, decaying exponentially within the same distance λ.

**Figure 7:**
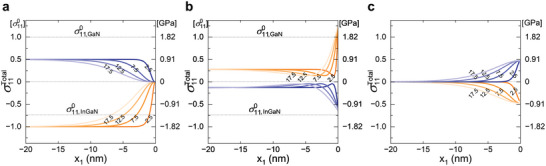
Stress fields calculated for various layer period λ. Four different λ, 2.5, 7.5, 12.5, and 17.5 nm with the constant InGaN/GaN thickness ratio of 0.5, are considered. Total stress profiles $\sigma^{\mathrm{Total}}=\sigma^{0}+\sigma^{S}$ of a) radial stress component $\sigma_{11}^{Total}$, b) axial stress component $\sigma_{22}^{Total}$, c) shear stress component $\sigma_{12}^{Total}$. The stress profiles extracted from the center of InGaN ($x_{2}=0$) and GaN ($x_{2}=(t_{\mathrm{InGaN}}+t_{\mathrm{GaN}})2$) layer are displayed in orange and blue graded lines, respectively. The distance over which stress relaxes from the surface increases as the layer period λ becomes larger.

The analysis using the elastic continuum model shows that the distance over which stress relaxes from the surface increases as the layer period ($\lambda =t_{\mathrm{InGaN}}+t_{\mathrm{GaN}}$) becomes larger (**Figure** **7**). This results in a smoother gradient in the stress profile, as the thicker layers facilitate a more gradual transition of stress throughout the heterostructure. Consequently, the undulation in the axial stress distribution also shifts further away from the surface as λ increases. Overall, a larger λ promotes more gradual stress relaxation.

**Figure 8:**
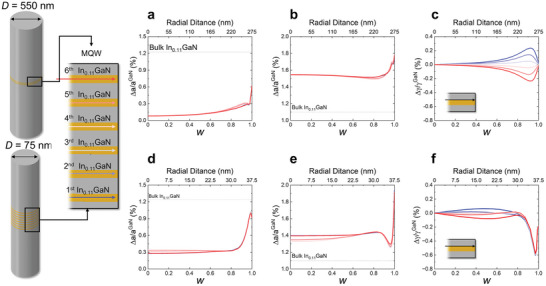
Effect of wire diameter (*D*) on the elastic strain relaxation of InGaN/GaN MQWs simulated by FEM. The radial distribution of the lattice misfit strain of all six In_0.11_GaN layers in the InGaN/GaN MQWs is plotted from the wire center (*w* = 0) to the sidewall (*w* = 1). a,d) In‐ plane, b,e) out‐of‐plane, and c,f) shear strain of the wire with *D* = 550 and *D* = 75 nm, respectively. The thicknesses of GaN and InGaN layers in the MQWs and the height of wires remain the same in the two model systems. The strain partitioning and relaxation in the InGaN/GaN MQWs of the 75 nm‐thick wire is more pronounced than in the 550 nm‐thick wire. As a consequence, InGaN QW‐to‐QW variation of the normal strain is larger and the shear strain propagates deeper into the central region of the 75 nm‐thick wire.

It is important to note that this analysis assumes a wire diameter *D* significantly larger than the periodicity λ and the total height *h* of multilayer structure. If the wire diameter becomes smaller and approaches roughly 10 λ and/or the total height *h* becomes comparable to the wire diameter *D*, each InGaN layer will be experiencing varying extent of the image stress due to the increasing role of strain relaxation that penetrates deeper into the central region, leading to more complex strain distribution between each layer.^[^ ^21^ ^]^ This is because, in the wire with a smaller diameter, mechanical constraints due to the traction‐free sidewalls are reached more swiftly. For example, the FEM simulation conducted for the same InGaN/GaN MQW structure inserted in a 75 nm‐wide wire shows that both the radial and axial strain induced in each layer of the MQW are reduced due to more pronounced strain partitioning but diverge noticeably from layer to layer even in the central region of wire (*w* = 0), a characteristic which is not pronounced in the 550 nm‐wide wire (**Figure** **8**). Additionally, the shear strain that facilitates elastic strain relaxation at the sidewall exhibits a different behavior depending on the wire diameter. In the 550 nm‐wide wire, the shear strain is mostly confined near the surface with opposite sign (Figure 8c). However, in the central region of 75 nm‐wide wire, the internal shear stress is induced noticeably due to this diverging normal strain. Moreover, the shear strain with the same negative sign is observed near the surface at 0.9 *w* (Figure 8f). These findings indicate that as the wire diameter decreases, controlling the strain in the MQW becomes increasingly complex, nonuniform distribution between each layer, complicating the strain control of MQW. Such nonuniform strain distribution within each layer of MQW significantly affects the µLED performance by means of strain‐induced polarization.

### 2.4 Piezoelectric Polarization

Understanding the piezoelectric polarization ($P^{\mathrm{PZ}}$) in a µLED is crucial, as the strain relaxation within the MQW structure markedly influences the piezoelectric properties of the active material. The $P^{\mathrm{PZ}}$ is inherently linked to strain, especially in the wurtzite crystal structures like InGaN/GaN, where lattice strain directly affects the electric field within the materials. The strain induced along the [0001] direction, being the primary polar axis in this wurtzite crystal structure, is particularly significant in the context of piezoelectric effects.^[^ ^1^, ^5^ ^]^

The influence of strain relaxation on piezoelectric polarization ($P^{\mathrm{PZ}}$) has been a subject of considerable interest in the study of various InGaN/GaN MQW structures.^[^ ^3^, ^23^, ^35^ ^]^ To assess this relationship, we utilized the experimental 2D strain maps, employing them to calculate the $P^{\mathrm{PZ}}$ along the [0001] direction. This calculation follows an established relationship, as outlined in the reference:^[^ ^36^ ^]^

(8) $$P_{2}^{PZ}=2e_{21}\varepsilon_{11}+e_{22}\varepsilon_{22}$$

where $\varepsilon_{\mathrm{ij}}$ and $e_{\mathrm{ij}}$ represent the strain and the piezoelectric coefficient, respectively. The $\left[1\bar{1}00\right]$ and [0001] direction correspond to the *x*_1_ and *x*_2_ directions, respectively. The values used in this calculation are: $e_{22}=73\;\;\mu C/{\mathrm{cm}}^{2}$, $e_{21}=-34\;\;\mu C/{\mathrm{cm}}^{2}$ for GaN, and $e_{22}=76\;\;\mu C/{\mathrm{cm}}^{2}$, $e_{21}=-37\;\;\mu C/{\mathrm{cm}}^{2}$ for InGaN as discussed in literature.^[^ ^36^ ^]^ According to the Gauss's law, $\nabla \cdot P_{2}^{PZ}=-\rho$, where ρ represents the charge density, a gradient of $P_{2}^{PZ}$ across the InGaN/GaN interface results in the sheet charges at the interface.

**Figure 9:**
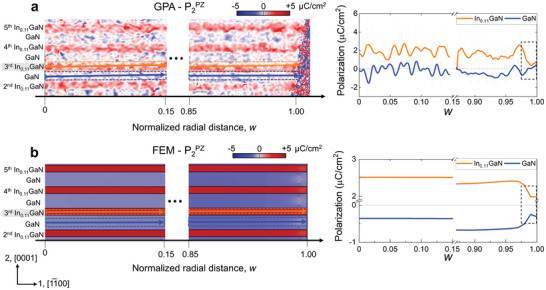
Piezoelectric polarization calculated from the STEM GPA strain maps and FEM strain maps. The out‐of‐plane piezoelectric component $P_{2}^{PZ}$ along the [0001] direction calculated using a) STEM‐GPA strain maps and b) FEM strain maps. For each data set the $P_{2}^{PZ}$ maps in the central region (0 < *w* < 0.15) and near‐surface zone (0.85 < *w* < 1.00) close to sidewall are shown. Note that the overall $P_{2}^{PZ}$ distribution follows the tendency of the out‐of‐plane strain map due to the more significant contribution of ≈$e_{22}$ to $P_{2}^{PZ}$ compared with $e_{21}$. In the near‐surface zone of the sidewall, the intricate strain state – characterized by diverging normal strains, alternating shear strains, and volume expansion due the elastic relaxation – leads to decreases in the gradient of $P_{2}^{PZ}$ across the InGaN/GaN interface, reducing the bound charges.

The $P_{2}^{PZ}$ maps and profiles obtained by experiment and FEM are shown in **Figure** **9**A,B, respectively. The overall $P_{2}^{PZ}$ distribution follows the tendency of the out‐of‐plane strain map due to the more significant contribution of $e_{22}$ to $P_{2}^{PZ}$ compared with $e_{21}$. In the central region, the alternating tensile and compressive strain of InGaN and GaN along the *x*_2_ direction is attributed to the opposite sign of $P_{2}^{PZ}$, resulting in the average $P_{2,InGaN}^{PZ}=2.5\;\mu C/cm^{2}$ and $P_{2,GaN}^{PZ}=-0.4\;\mu C/cm^{2}$ from FEM, $P_{2,InGaN}^{PZ}=2.1\;\mu C/cm^{2}$ and $P_{2,GaN}^{PZ}=-0.8\;\mu C/cm^{2}$ from experiments, respectively. This indicates the presence of a polarization gradient across the InGaN/GaN interface, which leads to the formation of sheet charges at the interface. Compared to a planar InGaN/GaN MQW structure, the strain partitioning in the central region of the µLED reduces both the polarization gradient and the interface sheet charges, is beneficial for reducing the piezoelectric field.^[^ ^3^, ^23^ ^]^

However, in the near‐surface zone of the sidewall (0.85 < *w* < 1.00), where strain relaxation is more pronounced, the intricate strain state – characterized by diverging normal strains, alternating shear strains, and volume expansion due the elastic relaxation – leads to decreases in the gradient of $P_{2}^{PZ}$ across the InGaN/GaN interface. The $P_{2}^{PZ}$ measured at the vicinity of sidewall are $P_{2,\mathrm{InGaN}}^{\mathrm{PZ}}=0.3\;\;\mu C/{\mathrm{cm}}^{2}$ and $P_{2,\mathrm{GaN}}^{\mathrm{PZ}}=0.2\;\;\mu C/{\mathrm{cm}}^{2}$ (Figure 9a). While the corresponding values calculated from FEM ($P_{2,\mathrm{InGaN}}^{\mathrm{PZ}}=2\;\;\mu C/{\mathrm{cm}}^{2}$ and $P_{2,\mathrm{GaN}}^{\mathrm{PZ}}=-0.2\;\;\mu C/{\mathrm{cm}}^{2}$) are slightly different, the trend of the radial variation of $P_{2}^{PZ}$ remains closely similar (Figure 9b). Notably, the reduced polarization gradient near the surface decreases the associated bound charges, and lowers the potential barrier. This potentially contributes to the formation of preferred carrier paths and causes spectral broadening in the near‐surface sidewall regions.^[^ ^9^, ^11^, ^21^, ^39^ ^]^

**Figure 10:**
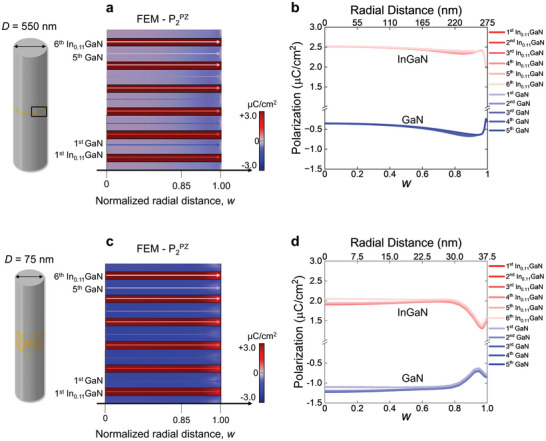
Effect of wire diameter (*D*) on the strain‐induced piezoelectric polarization of InGaN/GaN MQWs simulated by FEM. Piezoelectric polarization ($P_{2}^{\mathrm{PZ}}$) maps and profiles along the radial direction of the wire with a,b) *D* = 550 nm and c,d) *D* = 75 nm. As the diameter of the wire is reduced from 550 nm to 75 nm, due to the enhanced strain partitioning the $P_{2}^{\mathrm{PZ}}$ of the InGaN QW decreases but that of GaN QB increases compared to those of 550 nm‐wire. The $P_{2}^{\mathrm{PZ}}$ of the InGaN layers in the 75 nm‐wire exhibits nonuniform distribution between the layers.

As the diameter of µLED shrinks to the nanoscale, the impact of elastic relaxation from the sidewall on piezoelectric polarization $P_{2}^{PZ}$ becomes increasingly significant. To explore the size effects, we calculated the $P_{2}^{PZ}$ of the two cylindrical LEDs with different diameters using the simulated strain results via FEM (Figure 8), as depicted in **Figure** **10**. As can be expected from the strain distribution given in Figure 8, the $P_{2}^{PZ}$ of the InGaN layers in the smaller wire (75 nm‐wide diameter with *h*/*D* = 0.6) exhibits a noticeable layer‐to‐layer divergence (Figure 10). For instance, in the central region (0–0.2 *w*), the $P_{2}^{PZ}$ of the InGaN layers in the 550 nm‐wide wire is uniform with an average value of $P_{2,\mathrm{InGaN}}^{\mathrm{PZ}}=2.5\;\;\mu C/{\mathrm{cm}}^{2}$ (Figure 10b), showing no clear layer‐to‐layer divergence. However, in the 75 nm‐wide wire, $P_{2}^{PZ}$ shows a distinct layer‐to‐layer divergence, where $P_{2,6^{\mathrm{th}}-\mathrm{InGaN}}^{\mathrm{PZ}}$ and $P_{2,1^{\mathrm{st}}-\mathrm{InGaN}}^{\mathrm{PZ}}$ are $2.05\;\;\mu C/{\mathrm{cm}}^{2}$ and $1.9\;\;\mu C/{\mathrm{cm}}^{2}$, respectively (Figure 10d). This layer‐to‐layer divergence of $P_{2}^{PZ}$ becomes weaker as it moves toward the surface

Another notable feature is the rate of change in $P_{2}^{PZ}$ with the radial distance of wire. For the 550 nm‐wide wire, there is an abrupt change in $P_{2}^{PZ}$ ≈ 0.98–0.99 *w*, which we attribute to the multiplicative factor related to the periodicity λ, as discussed in Section 2.3. In contrast, for the 75 nm‐wide wire, the comparable length scale of the MQW and wire diameter (*h*/*D* = 0.6) results in a monotonic relaxation of normal strain already from approximately 0.8 *w*, although a rapid undulation is still observed from 0.94 *w*. Consequently, the $P_{2}^{PZ}$ of both InGaN and GaN layers starts decreasing toward each other from 0.8 *w* and then diverging from 0.94 *w* (Regardless of the ratio between λ and *D*, the undulation within the length scale of λ appears to be intrinsic to the periodicity itself). Although the gradient of $P_{2}^{PZ}$ becomes reduced across the InGaN/GaN interfaces in the smaller wire due to the pronounced elastic relaxation, the fluctuation of $P_{2}^{PZ}$ over an extended radial distance (≈20% of wire diameter) can cause severe spectral broadening and peak shift of emitted light.

The strain distribution identified in this study is likely prevalent in other epitaxial axial heterostructures and become increasingly critical as the dimensions of these structures shrink to the nanoscale.^[^ ^37^, ^38^ ^]^ The resultant complex strain fields, morphological changes, and associated strain‐induced piezoelectric polarization near the surface can significantly alter the physical, electrical, and optical properties of these systems. For µLEDs, where surface emission plays a dominant role, this interface‐constrained strain relaxation is particularly crucial as it impacts spectral broadening and wavelength shifts in emitted light.^[^ ^9^, ^11^, ^21^ ^]^

Furthermore, the elastic relaxation of InGaN/GaN MQWs, which occurs during the etching process, is likely linked to the formation of etching‐induced defects.^[^ ^18^, ^32^ ^]^ The elastic relaxation can promote the formation of defects such as Shockley–Read–Hall (SRH) centers by lowering the formation energy of surface defects.^[^ ^40^, ^41^ ^]^ These defects serve as non‐radiative recombination centers, contributing to increased surface leakage current and reduced external quantum efficiency. In combination with etching‐induced damage, the elastic relaxation plays a significant role in this degradation. While post‐etching surface passivation can remedy some of these defects, this underscores the importance of optimizing the etching and passivation processes to minimize their impact.

## 3 Conclusion

In this study, we have investigated the elastic relaxation of misfit strain near the sidewall of InGaN/GaN MQW heterostructure within µLEDs through experimental strain mapping and FEM simulations. Our analysis focused on a µLED with an *h/D* ratio of 0.08 (*h* = 45 and *D* = 550 nm). In this geometrical configuration, the misfit strain is partitioned between the InGaN QW and GaN QB, leading to opposite pre‐stress ($\sigma^{0}$) acting in each layer. The elastic relaxation of these complementarily strained layers toward their bulk state becomes pronounced from approximately 0.9 *w*. At the sidewall, the requirement for compensating the image stress ($\sigma^{S}$) to counterbalance the zero‐traction condition results in a complex stress distribution. The influence of image stress, characterized by periodic traction at the sidewall with a periodicity of λ and an exponentially decaying internal stress, necessitates the development of interfacial shear stresses near the surface. This shear stresses, appearing at both upper and lower interfaces of each layer with opposite signs, serve to counterbalance the elastic relaxation while maintaining lattice coherency. Additionally, the axial normal stress near the surface is notably amplified due to counteracting the traction, leading to a marked volume expansion in the InGaN QW layers. The characteristic distance (λ = 7.5 nm ≈0.03 *w*) aligns well with our experimental observations and FEM results, where effective elastic relaxation begins from ≈0.97 *w*.

## 4 Experimental Section

### Fabrication of µLEDs

For the fabrication of µLEDs, a (0001)‐oriented 4‐inch *n*‐type GaN/sapphire wafer pre‐structured with six pairs of In_0.11_GaN/GaN MQWs was used. The thickness of each InGaN QW and GaN QB was 2.5 and 5 nm, respectively. Followed by growth of *p*‐type GaN, an indium tin oxide (ITO) layer was deposited, which serves as the transparent conducting electrode. A regular array of µLED pattern with the wire diameter of ≈550 nm with the length of 4 µm was fabricated through photolithography followed by reactive ion etching. Subsequently, potassium hydroxide (KOH) wet etching was performed to remove the sidewall damage caused by the plasma during the reactive ion etching.

### TEM Specimen Preparation

Specimens for (S)TEM‐GPA strain mapping were prepared by using a focused ion beam (FIB, Helios 5UX, Thermofisher). The FIB milling process consists of a series of carefully controlled thinning steps. Initially, the specimens were thinned down to approximately 500 nm using a Ga^+^ ion beam with the beam of 0.26 nA at the acceleration voltage of 30 kV. This initial rough milling was followed by a further thinning and cleaning step at the beam of 90 pA at the acceleration voltage of 5 kV. The final polishing was carried at the beam of 41 pA at the acceleration voltage of 5 kV. A different strategy was adopted to prepare a TEM specimen suitable for assessing the pristine state of the surface morphology of µLED, which do not employ any thinning process. The sapphire substrate with vertical array of µLED was cut into an ∼500 µm thick slice using a low‐speed diamond saw. In order to prevent contamination of the surface of µLED during cutting, no lubricating or cooling agent was used. After the dry cutting of the substrate, the µLEDs were observed to exist stable on the substrate without being damaged or contaminated. The slice was subsequently affixed to a half‐circle Cu grid using a silver epoxy. The STEM image obtained from this TEM specimen is presented in Figure 1d (right one).

### Scanning Transmission Electron Microscopy (STEM)

HAADF STEM images were acquired using a Cs‐corrected STEM (JEM‐ARM200F, Jeol Lt. Tokyo, Japan) operated at 200 kV. The probe convergence angle of 21.5 mrad was used for HAADF STEM imaging with the collection angle of 80 – 220 mrad. Images were obtained with 2048 × 2048 pixels and an acquisition time of 2 µs per pixel. The background noise of image was removed by local 2D Wiener filtering from a commercial software (HREM‐Filters Pro, HREM Research Inc., Japan).

### Geometric Phase Analysis of STEM Images for Strain Mapping

Individual high‐resolution HAADF STEM images obtained from different regions of µLED were stitched to apply the same reference for strain analysis. For the GPA strain mapping of HAADF STEM images, a commercially available software (GPA, HREM Research Inc., Japan) was utilized.^[^ ^42^ ^]^ The diffraction spots corresponding to $d_{\left(1\bar{1}00\right)}$ and *d*_(0002)_ lattice planes were selected with a virtual mask size of 1 nm^−1^ for the in‐plane and out‐of‐plane strain mapping, respectively. This size mask was chosen to balance the spatial resolution and signal‐to‐noise ratio of the strain map. The geometric phase of the selected diffraction spots was calculated and used for strain mapping. The geometric phase of *n*‐type GaN in the central region was selected as the reference for strain mapping.

### Finite Element Analysis (FEA) Simulation

The finite element analysis (FEA) method was employed to examine the deformation field within µLED that possesses MQW. The commercial software ABAQUS was utilized as the solver for the FEA simulation. To accurately model the lattice mismatch between the materials, the simulation was executed in two stages. Initially, the In_0.11_GaN layers, having a greater lattice constant relative to GaN, were compressed to align with the GaN lattice constant. Subsequent to this adjustment, constraints were applied to ensure that displacement and electric potential are consistent across the interface between the GaN and In_0.11_GaN layers and remove the boundary condition for compression to relax it. Material properties for In_0.11_GaN were determined by interpolating between those of GaN and InN. The elastic stiffness tensor utilized in FEA calculation for GaN and In_0.11_GaN are shown in Table S1 (Supporting Information). To enhance the accuracy and computational efficiency of the FEA further, a two‐level meshing approach was employed in this study. In areas distant from the MQW, where stress variation was less significant, a coarse mesh was applied to alleviate the computational load. Conversely, a finer mesh was utilized in the vicinity of the MQW, where stress variation was more pronounced, ensuring that local deformation behavior was captured with higher precision.

## Conflict of Interest

The authors declare no conflict of interest.

## Author Contributions

J.K. and J.Y. contributed equally to this work. S.H.O. conceptualized the whole project and the experiments. J.K. and B.P. performed the TEM experiments and analysis. J.Y. and S.R. performed FEA simulations and solved Airy stress function. GPA strain analysis were performed by B.P., J.K., and J.J under supervision of S.H.O. and the mechanical modelling by S.R., and J.Y. J.K. and J.Y. prepared the first draft under supervision of S.R. and S.H.O.

## Supporting information



Supporting Information

## Data Availability

The data that support the findings of this study are available from the corresponding author upon reasonable request.
