# Supplementary material for: Elastic Relaxation of Coherent InGaN/GaN Interfaces at the Microwire LED Sidewall
Source: Adv Sci (Weinh). 2025 Feb 26;12(19):2408736. doi: 10.1002/advs.202408736 (PMC12097021; doi:10.1002/advs.202408736)
Supplement: Supplementary file 1 — Supporting Information [file ADVS-12-2408736-s001.pdf]

ADVANCED SCIENCE
Open Access

Supporting Information

for *Adv. Sci.*, DOI 10.1002/advs.202408736

Elastic Relaxation of Coherent InGaN/GaN Interfaces at the Microwire LED Sidewall

Jongil Kim, Jinwook Yeo, Bumsu Park, Jeehun Jeong, Seunghwa Ryu and Sang Ho Oh**

Supporting Information

Elastic Relaxation of Coherent InGaN/GaN Interfaces at the Microwire LED Sidewall

Jongil Kim, Jinwook Yeo, Bumsu Park, Jeehun Jeong, Seunghwa Ryu, Sang Ho Oh**

Table S1. Elastic stiffness tensor utilized in FEA calculation for GaN and $In_{0.11}GaN$.

Tensor Matrix

$$\begin{pmatrix} C_{11} & C_{12} & C_{13} & 0 & 0 & 0 \\ C_{12} & C_{22} & C_{23} & 0 & 0 & 0 \\ C_{13} & C_{23} & C_{33} & 0 & 0 & 0 \\ 0 & 0 & 0 & C_{44} & 0 & 0 \\ 0 & 0 & 0 & 0 & C_{55} & 0 \\ 0 & 0 & 0 & 0 & 0 & C_{66} \end{pmatrix}$$

GaN

$$\begin{pmatrix} 390 & 145 & 106 & 0 & 0 & 0 \\ 145 & 390 & 106 & 0 & 0 & 0 \\ 106 & 106 & 398 & 0 & 0 & 0 \\ 0 & 0 & 0 & 105 & 0 & 0 \\ 0 & 0 & 0 & 0 & 105 & 0 \\ 0 & 0 & 0 & 0 & 0 & 125 \end{pmatrix}$$

$In_{0.11}GaN$

$$\begin{pmatrix} 370 & 141 & 107 & 0 & 0 & 0 \\ 145 & 370 & 107 & 0 & 0 & 0 \\ 107 & 107 & 377 & 0 & 0 & 0 \\ 0 & 0 & 0 & 96 & 0 & 0 \\ 0 & 0 & 0 & 0 & 96 & 0 \\ 0 & 0 & 0 & 0 & 0 & 114 \end{pmatrix}$$

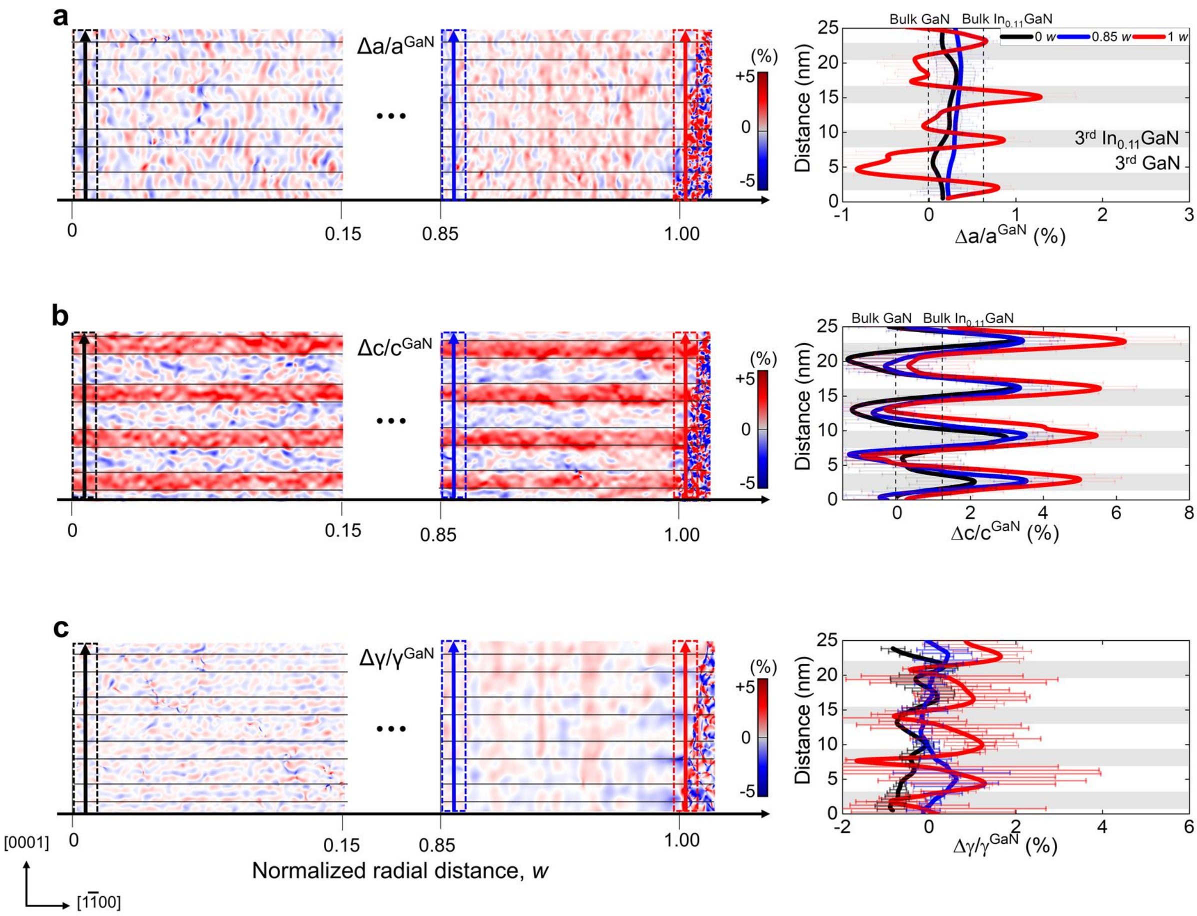

Figure S1. STEM GPA strain maps and profiles drawn across the InGaN/GaN MQWs at three different locations along the radial direction. a) In-plane, b) out-of-plane and c) shear strain maps showing distribution of strain at the central region ($0 < w < 0.15$) and the near surface region ($0.85 < w < 1$). The strain pattern at the central region ($w = 0$), which is characterized by strain partitioning between InGaN and GaN, remains almost the same up to $w = 0.85$. Near the surface ($w = 1$), the elastic relaxation reverts the lattice parameters of each layer to the corresponding bulk values. Note that both in-plane and out-of-plane lattice parameters of InGaN QW increases beyond its bulk value, leading to volume expansion. In the shear strain maps and profiles, opposite sign of interfacial shear strain evolves across each interface near the surface region.

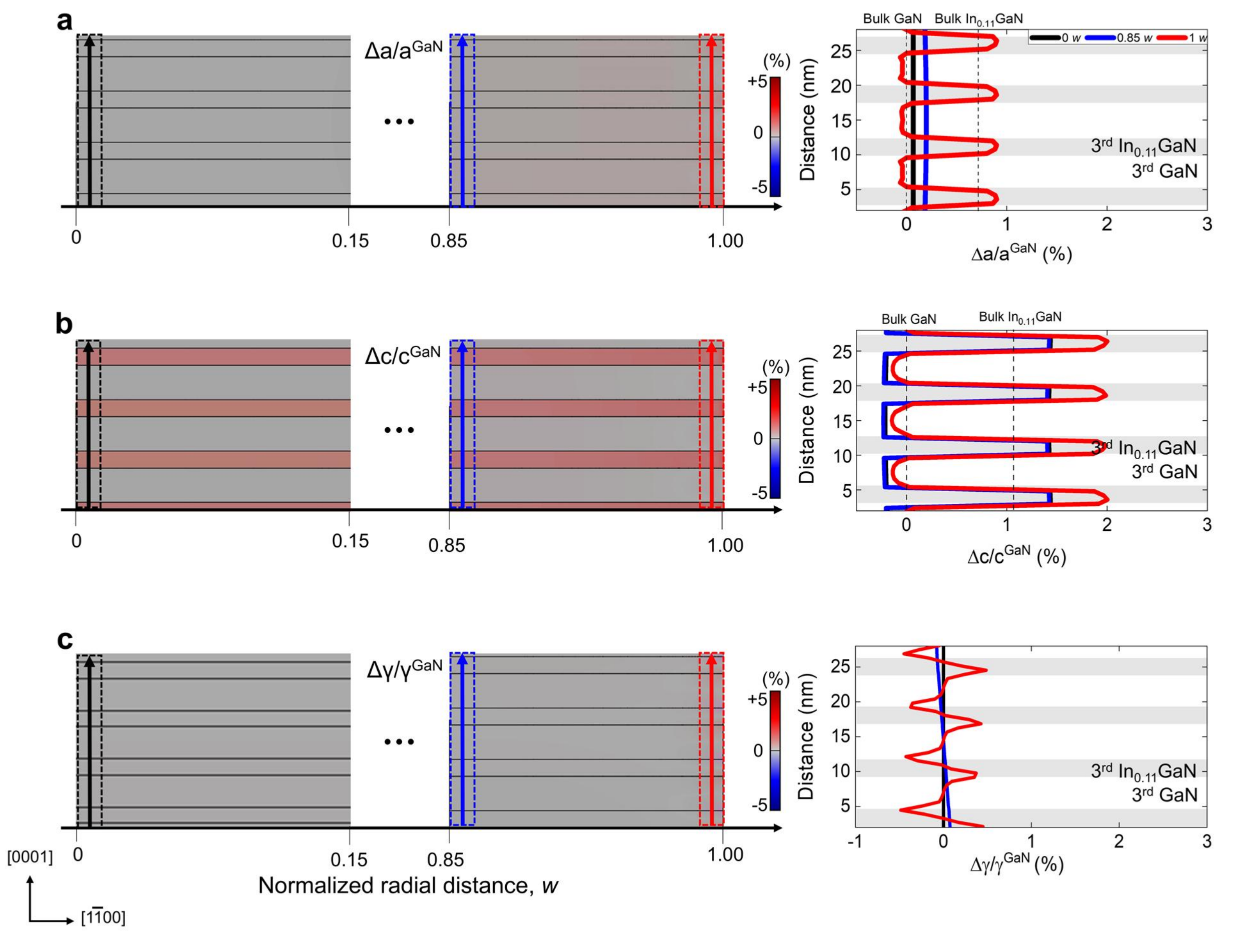

Figure S2. FEM simulation strain maps and profiles drawn across the InGaN/GaN MQWs at three different locations along the radial direction. Presented in the same way as in Figure S1 for comparison. Experimentally observed strain distribution is perfectly reproduced by FEM.

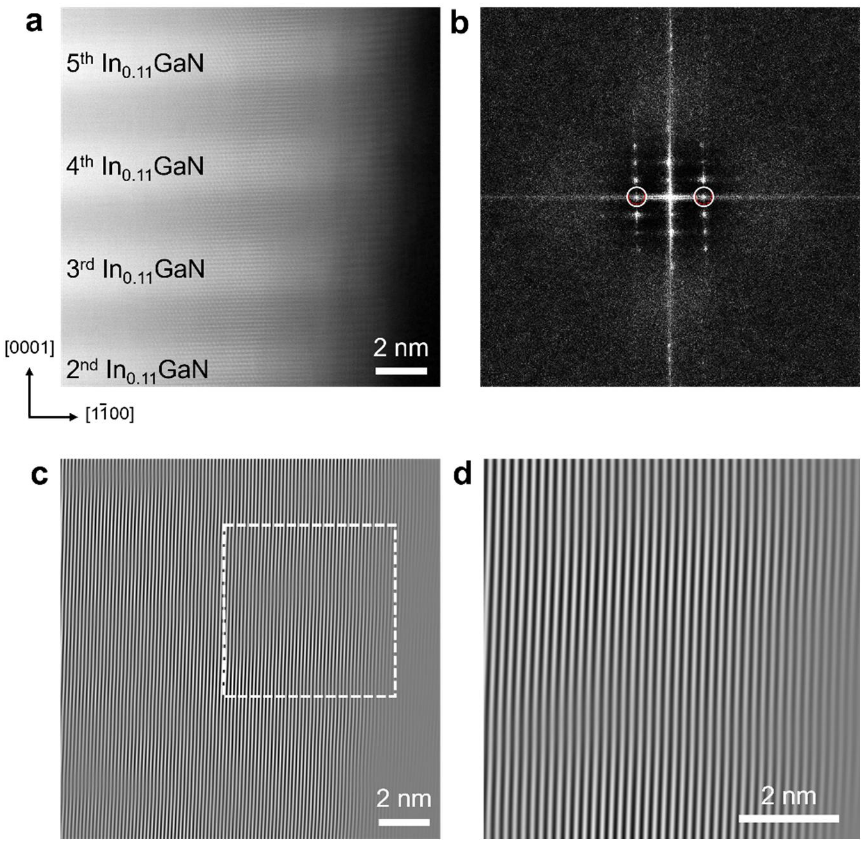

Figure S3. HAADF STEM and corresponding inverse fast Fourier transform (IFFT) images showing lattice coherency across the interface at the μLED sidewall. a) HAADF STEM image of the μLED sidewall shown in Figure 1D. b) Corresponding FFT pattern. White circles indicate the selected in-plane reflections for IFFT image depicted in c). d) Magnified view of IFFT image from the area indicated by dashed white line. Lattice coherency of $In_{0.11}GaN/GaN$ is maintained all the way down to the traction-free surface.

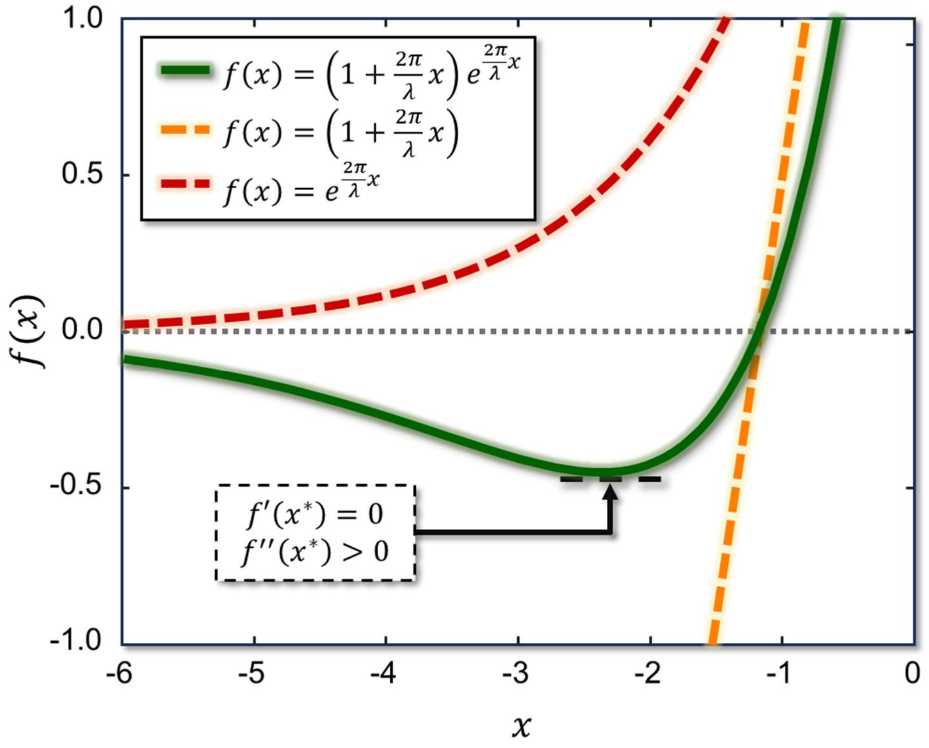

Figure S4. Comparison of polynomial function $(1+\frac{2\pi}{\lambda}x)$, exponential function $\exp(\frac{2\pi}{\lambda}x)$, and their combined function $(1+\frac{2\pi}{\lambda}x)\exp(\frac{2\pi}{\lambda}x)$. The combined function exhibits concavity around a critical point x^*, where its first derivative equals to zero $(f'(x^*)=0)$. This results in a non-monotonic characteristic, with the function decreasing before x^* and increasing afterward, highlighting a transition in its behavior at this point.
